# Inactivation of APC Induces CD34 Upregulation to Promote Epithelial-Mesenchymal Transition and Cancer Stem Cell Traits in Pancreatic Cancer

**DOI:** 10.3390/ijms21124473

**Published:** 2020-06-23

**Authors:** Mei Jen Hsieh, Tai-Jan Chiu, Yu Chun Lin, Ching-Chieh Weng, Yu-Ting Weng, Chang-Chun Hsiao, Kuang-hung Cheng

**Affiliations:** 1 Institute of Biomedical Sciences, National Sun Yat-Sen University, Kaohsiung 80424, Taiwan; rohsiehs@yahoo.com.tw (M.J.H.); yoyo32201@yahoo.com.tw (Y.C.L.); inoursky@gmail.com (C.-C.W.); nina2398a@gmail.com (Y.-T.W.); 2 Division of Neurology, Department of Internal Medicine, Kaohsiung Armed Forces General Hospital, Kaohsiung 802, Taiwan; 3 Division of Hematology/Oncology, Department of Internal Medicine, Chang Gung Memorial Hospital, Chang Gung University, Kaohsiung 83301, Taiwan; kuerten@cgmh.org.tw; 4 Graduate Institute of Clinical Medical Sciences, College of Medicine, Chang Gung University, Kaohsiung Chang Gung Memorial Hospital, Kaohsiung 83301, Taiwan; 5 National Institute of Cancer Research, National Health Research Institutes, Tainan 704, Taiwan; 6 Department of Medical Laboratory Science and Biotechnology, Kaohsiung Medical University, Kaohsiung 80708, Taiwan; 7 Regenerative Medicine and Cell Therapy Research Center, Kaohsiung Medical University, Kaohsiung 80708, Taiwan

**Keywords:** pancreatic ductal adenocarcinoma (PDAC), APC, CD34, cancer stemness, metastasis

## Abstract

Pancreatic cancer (PC) is a highly lethal malignancy due to the cancer routinely being diagnosed late and having a limited response to chemotherapy. Pancreatic ductal adenocarcinoma (PDAC) is the most common form of pancreatic malignant tumor, representing more than 85% of all pancreatic cancers. In the present study, we characterized the phenotypes of concomitant P53 and APC mutations in pancreatic neoplasms driven by the oncogene KRAS in genetically modified mice (GEMM). In this GEMM setting, APC haploinsufficiency coupled with P53 deletion and KRAS^G12D^ activation resulted in an earlier appearance of pancreatic intraepithelial neoplasia (PanIN) lesions and progressed rapidly to highly invasive and metastatic PDAC. Through a microarray analysis of murine PDAC cells derived from our APC-deficient PDAC model, we observed that APC loss leads to upregulated CD34 expression in PDAC. CD34 is a member of a family of single-pass transmembrane proteins and is selectively expressed in hematopoietic progenitor cells, vascular endothelial cells, interstitial precursor cells, and various interstitial tumor cells. However, the functional roles of CD34 in pancreatic cancer remain unclear. Thus, in this study, we explored the mechanisms regarding how CD34 promotes the deterioration of pancreatic malignancy. Our results demonstrated that the increased expression of CD34 induced by APC inactivation promotes the invasion and migration of PDAC cells, which may relate to PDAC metastasis in vivo. Collectively, our study provides first-line evidence to delineate the association between CD34 and the APC/Wnt pathway in PDAC, and reveals the potential roles of CD34 in PDAC progression.

## 1. Introduction

Pancreatic cancer (PC) is the fourth most common cause of adult cancer mortality and is recognized as the most lethal human cancer worldwide [1]. Pancreatic ductal adenocarcinoma (PDAC) is the most common lethal PC, with fewer than 8% of newly diagnosed patients surviving longer than five years. Despite numerous clinical trials evaluating new agents and treatment combinations, PDAC remains highly refractory to treatment. The highly invasive and chemoresistant nature of PDAC are the reasons for poor prognosis [2,3,4]. Several studies reported smoking and long-standing chronic pancreatitis as clear risk factors; diabetes and obesity also appear to confer increased risks [5,6,7]. On the genetic level, several studies documented an increased risk in relatives of PDAC patients; it is estimated that 10% of pancreatic cancers are due to an inherited predisposition, for instance, germline mutations in the BRCA1/2 or LKB1 genes [8]. The obviously molecular alterations in PDAC include multiple evolutionary steps of the precursor lesions, whereby progression involves the acquisition of mutations in KRAS, INK4a, P53, SMAD4, APC, or β-catenin. More recently, studies recognized that PDAC can develop from three distinct types of precursor lesion that affect the pancreatic ducts, namely, pancreatic intraepithelial neoplasms (PanINs), intraductal papillary mucinous neoplasms (IPMNs), and mucinous cystic neoplasms (MCNs) [9,10,11,12]. Most of these precursor lesions are benign and noninvasive, but patients with these lesions demonstrate a much higher risk of developing malignant PDAC within their lifetime.

Development of the pancreas is a highly coordinated process and is initiated by the outgrowth of dorsal and ventral pancreatic buds. The adult pancreatic exocrine mass also contains endocrine islet formation, which is regulated by several well-defined key transcription factors and signaling pathways [9]. The Wnt signaling pathway emerged as one of the critical signaling pathways, playing an important role in the development of many organs, including the pancreas [10]. Recently, a study showed that β-catenin signaling in dispensable in controlling pancreas organ growth and shape but may be required for pancreas regeneration. The APC gene was first characterized as a crucial tumor suppressor gene of the distal gastrointestinal tract, with germ-line mutations in APC resulting in familial adenomatous polyposis [13,14]. Somatic mutations in APC are frequently observed in rectal tumors, however, only rare mutations are reported in PDAC. It is notable that somatic mutations in the APC gene are found more commonly in rare types of PC [15,16]. APC was shown to negatively regulate the Wnt/β-catenin pathway and promote cytosolic β-catenin ubiquitination and degradation. Inactivation of APC leads to increased activity of the canonical Wnt signal pathway in different cancers [17,18,19,20]. Recent studies demonstrated that the Wnt/β-catenin pathway is reactivated in PanINs and that its expression levels gradually increase during disease progression [21,22]. Notably, in addition to targeting β-catenin for degradation, APC is also involved in cell polarity and chromosome segregation [23,24]. Many studies of genetically modified APC-deficient mouse strains demonstrated that APC is crucial in colon, skin, breast, thymus, and nervous system development, as well as neoplasia production [25,26,27].

Genetically engineered mouse models (GEMMs) provide a valuable system to define genotype–phenotype relationships in many human tumors, including PDAC. In 2003, Nabeel and DePinho developed the first genetically engineered mouse model that expressed an oncogenic KrasG12D allele in the developing pancreas through Cre-mediated recombination driven by Pdx1 regulatory elements. This model recapitulated the multistep progression of the cognate human disease, including the presence of mouse PanIN (mPanINs) at various histological grades [28,29]. Recently, we continued to generate several PDAC mouse models to recapitulate the histopathologic progression and genetics of human PDAC [28]. A typical PDAC model was engineered to express Kras^G12D^ and delete P53 specifically in the pancreas by crossing with the Pdx1-Cre strain. Kras^G12D^ activation alone caused the development of PanINs of different grades which, combined with the loss of p53, produced a rapid progression to PDAC [29,30,31]. Notably, although the genetic lesions in this model were present in all pancreatic lineages, the resulting neoplastic changes showed an exclusively ductal phenotype, suggesting either that these mutant alleles drive transdifferentiation of multiple pancreatic cell lineages to a ductal character or that such lesions produce oncogenic change exclusively in the ductal progenitor lineage to dominate PDAC development [12,31,32].

CD34 is a member of a family of single-pass transmembrane proteins that is expressed in early hematopoietic and vascular-associated tissues [33,34]. It may also mediate the attachment of stem cells to the bone marrow extracellular matrix or directly to stromal cells. CD34 is also an important adhesion molecule and is required for T cells to enter lymph nodes [35,36]. It is expressed on lymph node endothelia, whereas the L-selectin molecule to which it binds is expressed on the T cell. Conversely, under other circumstances, CD34 was shown to act as molecular “Teflon” and block mast cells, eosinophils, and dendritic cell precursor adhesion and facilitate vascular lumen opening [37]. Recently, studies suggested that CD34 may also play a more selective role in chemokine-dependent migration of eosinophils and dendritic cell precursors [38]. Further, CD34 antigen staining was implicated as a prognostic marker of the microvascular count (MVC) in prostate cancer [39]. In this study, we found that inactivation of APC results in increased expression of CD34 in PDAC, and we hypothesized that the activation of the stem cell marker CD34 in PDAC contributes to the conversion of prometastatic and proangiogenic phenotypes of PDAC.

## 2. Results

### 2.1. Genetically Engineered Mouse Models (GEMMs) Recapitulate the Loss of APC in PDAC to Evoke Metastatic PDAC Development

**Figure 1:**
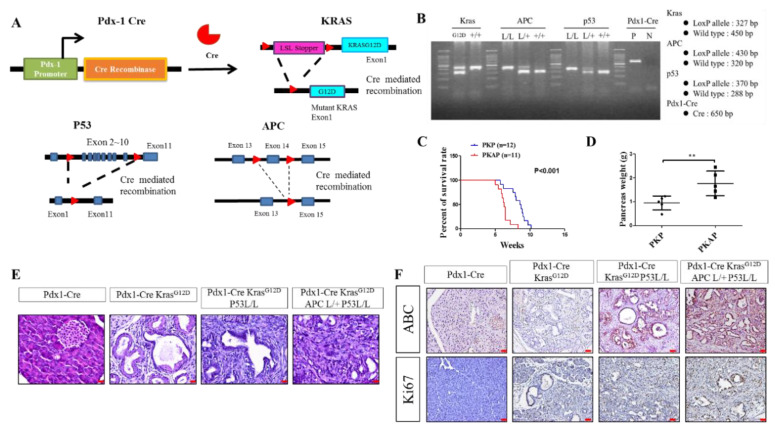
Modeling APC loss in pancreatic ductal adenocarcinoma (PDAC). (**A**) Illustration of strategies to generate Pdx1-Cre, Kras^G12D^, APC^L/L^, and p53^L/L^ (PKAP) mice. (**B**) Specific genotyping polymerase chain reaction (PCR) analysis for the detection of KRAS, APC, p53, and Pdx1-Cre target alleles from the mice tail DNA. (**C**) Kaplan–Meier curves showing the percentage of survival rates for mice of the indicated genotypes. (**D**) Data for mice showing pancreatic weight from the indicated genotypes. ** *p*-value < 0.01. (**E**) H&E-stained histological sections of pancreatic tissue from different genotypes of mice. (**F**) Immunohistochemical (IHC) staining demonstrated the expression levels of Ki67 and active β-catenin (ABC) in PDAC. The scale bar is 20 μm.

To investigate the effects of APC deletion on PDAC tumorigenesis, Pdx-1Cre Kras^G12D^ mutant mice were crossed with APC^CKO/CKO^ and p53-null mice to gain the Pdx1-Cre pancreas-specific transgenic allele in order to induce the activation of KrasG12D and specific deletion of APC and p53 in the pancreas (Figure 1A). Then, using polymerase chain reaction (PCR), we confirmed the deletion of APC, p53, and activation of the KrasG12D mutant allele mediated by pancreas-specific Cre-recombinase to organize the development of PDAC in mice, as shown in Figure 1B. In this setting, we observed that homozygous APC deletion resulted in early neonatal death. As reported previously, all Pdx1-CreKras^G12D^APC^CKO/+^p53^L/L^ (PKAP) mice (*n* = 20) developed a swollen abdomen with a palpable abnormal mass due to ascites fluid and pancreatic tumor burden within 6–10 weeks (Figure 1C) [31]. Pairwise log rank tests revealed that the average survival of Pdx1-CreKras^G12D^APC^CKO/+^p53^L/L^ mice was significantly shorter than that of the Pdx1-CreKras^G12D^p53^L/L^ (PKP) mice (Figure 1C). The average weight of the pancreata from PKAP mice was significantly heavier (*p*  <  0.01) than those of age-matched PKP (Figure 1D). Macroscopic examination showed that the neoplastic lesions arising from the Pdx1-CreKras^G12D^APC^CKO/+^ p53^L/L^ mice were a highly metastatic phenotype of PDAC (Figure 1E) [31]. Pdx1-CreKras^G12D^APC^CKO/+^p53^L/L^ mice developed metastatic PDAC harboring liver, lung, stomach, and colorectal metastases, confirmed by examining H&E stained sections; the representative histological sections for metastatic PDAC derived from PKAP are shown in Figure 1E. Additionally, IHC analysis revealed that Pdx1-CreKras^G12D^ APC^CKO/+^p53^L/L^ and Pdx1-Cre;Kras^G12D^APC^L/L^p53^L/L^ (PKAP) mice showed increased staining patterns of the cell proliferation protein Ki67 and active β-catenin (ABC) in the nucleus compared to that of Pdx1-CreKras^G12D^p53^L/L^ (Figure 1F). Taken together, APC deletion resulted in a rapid deterioration of pancreatic tumors into metastatic PDACs alongside the activation of Kras^G12D^ and p53 loss.

### 2.2. Inactivation of APC in PDAC Induced EMT to Increase PDAC Cell Tumorigenic and Migratory Abilities

**Figure 2:**
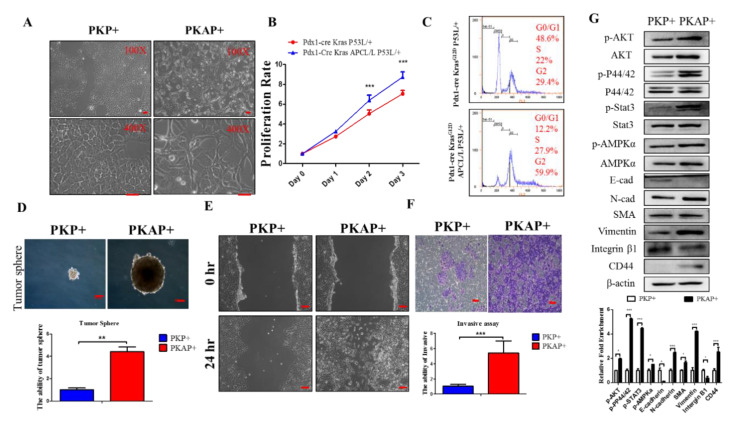
APC loss promotes tumorigenesis and cell motility in PDAC. (**A**) Cell morphology of primary murine PDAC cells from Pdx1-Cre; KrasG12D, p53L/+ (PKP+) mice and Pdx1-Cre, KrasG12D, APCL/L, and p53L/+ (PKAP+) mice. (**B**) The analysis of PKP+ and PKAP+ tumor cell proliferation rates by MTT assays. The results indicated that loss of APC promotes PDAC cell proliferation in vitro. (**C**) Representative flow cytometric analysis of cell cycle profiles from PKP+ and PKAP+ PDAC cells. Data are from three independent experiments. (**D**) Comparing PKP+ and PKAP+ tumor sphere formation by using sphere-forming assays. Inactivation of APC forms larger tumor spheres compared to PKP+ groups; *p* < 0.01 compared to PKAP+ with PKP+. The scale bar is 100 μm. (**E**) Loss of APC increases in vitro cell motility according to an in vitro wound healing assay. (**F**) An in vitro transwell invasion assay demonstrated that PKAP+ tumor cells have higher invasive ability than PKP+ tumor cell; *p* < 0.001 compared to PKAP+ with PKP+. Data are from three independent experiments. (**G**) Western blotting analysis revealed that APC loss increases the phosphorylation of PI3K, STAT3, and MAPK pathways and induces epithelial–mesenchymal transition (EMT) by comparing cell lysates between PKP+ and PKAP+ cells. Densitometric analysis of relative expression levels after normalization to loading control β-actin are presented in the lower panel.

Based on our PKAP mouse model findings, we speculated that the inactivation of APC may affect PDAC cell proliferation, migration, and invasion by activating a variety of downstream signaling pathways. Since homozygous deletions of both APC and p53 caused the most neonatal deaths, we harvested primary PDAC cells from Pdx1-CreKras^G12D^p53^L/+^ (PKP+) and Pdx1-CreKrasG12D APC^L/L^p53^L/+^ (PKAP+) mice for cellular physiological and downstream molecular analyses. Our results showed that PKP+ tumor cells exhibited a cuboidal cobblestone monolayer epithelial shape, while PKAP+ tumor cells displayed more spindle fibroblast-like morphology (Figure 2A). Further, using the cell proliferation MTT (3-[4,5-dimethylthiazol-2-yl]-2,5 diphenyl tetrazolium bromide) assay to compare the growth rates between these two different genotypes of PDAC cells, we observed that the proliferation rate of PKAP+ cells was significantly higher than that of PKP+ cells (Figure 2B). Intriguingly, using flow cytometry analysis to compare the cell cycle profiles of PKP+ and PKAP+ cells, we observed that most of the PKP+ cells were in the G1 phase, while the PKAP+ cells exhibited a higher portion of cells in the G2/M phase, which may have caused aneuploidy cells with abnormal chromosome numbers (>2N) (Figure 2C). Subsequently, using tumor sphere formation assays, we found that PKAP+ cells have greater tumor-forming abilities than PKP+ cells (Figure 2D). Furthermore, an in vitro cell scratch test was applied to evaluate in vitro cell migratory ability, revealing that PKAP+ tumor cells moved faster than PKP+ tumor cells and indicating that PKAP+ tumor cells may have greater cell migration abilities than PKP+ cells (Figure 2E). Similar conclusions were obtained from the transwell invasion assays (Figure 2F).

Epithelial cell transfer to a fibroblast-like morphology through a cell plasticity-promoting phenomenon known as epithelial–mesenchymal transition (EMT). EMT is a key mechanism of tumor metastasis, including in pancreatic tumors. During EMT in PDAC progression, expression of polarity and adhesion molecules on the surfaces of PDAC cells is decreased but expression of mesenchymal markers is induced to drive a highly invasive phenotype, leading to PDAC cells to detach from the primary tumor and disseminate to other distant organs. EMT is also a trait of cancer stemness properties, which are crucial for cancer recurrence, metastasis, and drug resistance. Our results obtained from Western blotting demonstrated that PKAP+ tumor cells reduced the expression of E-cadherin and integrin β1 and increased expression of N-cadherin, smooth muscle actin (SMA), and vimentin compared to PKP+ cells. Western blotting was also performed to detect changes in protein levels of the downstream signaling pathways between PKAP+ and PKP+ cells. We found that PKAP+ cells showed increased p-AMPKα, p-AKT, p-P44/42, and p-Stat3 protein levels when compared to PKP cells (Figure 2G). Additionally, as shown in Figure 2G, our data also showed increased expression of the cancer stem cell marker CD44 in PKAP+ cells compared to PKP+ cells. Taken together, our results demonstrated that the inactivation of APC in PDAC promotes cell proliferation and EMT and increases cancer cell migratory ability in vitro.

### 2.3. Activation of CD34 Pathway in PKAP+ PDAC Cells

**Figure 3:**
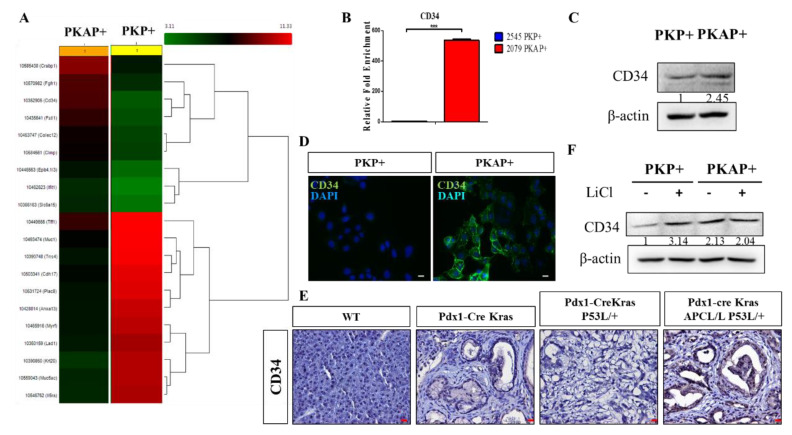
APC loss increases CD34 expression in PDAC. (**A**) Microarray analysis of primary PDAC cells derived from PKP+ and PKAP+ mice. (**B**) Confirmation of the mRNA expression levels of CD34 in PKP+ and PKAP+ cells using reverse real-time PCR. We found upregulation of CD34 gene expression in PKAP+ tumor cells. *** *p* < 0.001. (**C**) Immunoblotting analysis confirmation of increased CD34 protein expression in PKAP+ tumor cells compared to PKP+ cells. (**D**) Immunofluorescence staining (IF) showing significantly increased CD34 expression in PKAP+ tumor cells and its specific location in the cell membrane. Representative images are from three independent experiments. (**E**) Observation of CD34 expressed in tumor tissues by immunohistochemical (IHC) staining. (**F**) Treatment with 20 mM LiCl (Wnt pathway agonist) stimulates CD34 protein expression in PDAC. Fold change with respect to control untreated cells normalized to β-actin expression is depicted as numbers below each lane.

According to our above experiments, we demonstrated that PKAP+ cells exhibited significantly increased tumorigenic and cell migratory abilities. Subsequently, we performed the cDNA microarray approach to analyze the differential gene expression profiles between PKP+ and PKAP+ cells (Figure 3A). Intriguingly, we identified significantly increased CD34 gene expression in PKAP+ cells when compared to PKP+ cells through our cDNA microarray results. RT-q-PCR analysis was performed to confirm the increased CD34 gene expression in PKAP+ cells (Figure 3B). Similar results were obtained from Western blotting analysis to confirm increased protein expression of CD34 in PKAP+ tumor cells compared to PKP^+^ cells (Figure 3C). Immunofluorescence (IF) staining was further performed to investigate the cellular location and expression of CD34 in PDAC cells. We demonstrated that CD34 was abundantly expressed on the cell surface of PKAP+ tumor cells, as shown in Figure 3D. Meanwhile, using IHC staining, we also found upregulated CD34 protein expression in PDAC tissues derived from PKAP+ PDAC mice compared to PKP+ mice, and confirmed that CD34 was located in the cell membrane and cytoplasm of PDAC lesions in PKAP+ mice, as shown in Figure 3E. Thus, we concluded that the expression levels of CD34 in PKAP+ PDAC tissues were significantly higher than PKP+ tumors, implying that the inactivation of APC may lead to induced CD34 expression in PDAC. We also noted that PKP+ PDAC cells significantly increased CD34 gene expression after induction of the Wnt pathway activator LiCl (Figure 3F). Taken together, we hypothesized that the APC/Wnt pathway may play a crucial role in the regulation of CD34 expression in PDAC.

### 2.4. Knockdown of CD34 in PKAP+ Tumor Cells Reduces Cell Invasion and Migration

**Figure 4:**
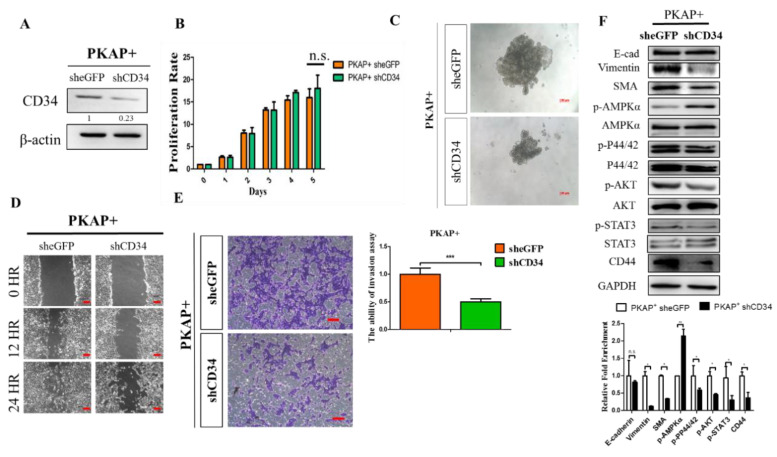
Knockdown of CD34 decreases in vitro tumorigenic potential and migratory capacities in PDAC. (**A**) Western blotting analysis confirmed the knockdown efficacy of CD34 in PKAP+ PDAC cells. (**B**) An MTT cell proliferation assay showed that knockdown of CD34 does not affect PKAP+ tumor cell proliferation rate in vitro. (**C**) Sphere formation assay determined that knockdown of CD34 significantly reduces sphere formation in PKAP+ tumor cells. (**D**) Knockdown of CD34 in PKAP+ PDAC cell reduces cell motility by using in vitro wound healing assay. (**E**) In vitro transwell invasion assay demonstrated that knockdown of CD34 reduces invasive ability in PKAP+ cell *p* < 0.001 compared to shCD34 with the sheGFP control. Data are from three independent experiments. (**F**) Western blotting indicated that knockdown of CD34 reverses EMT and downregulates cancer stemness marker CD44 but upregulates p-AMPKα protein level in PKAP+ PDAC cells. Densitometric analysis of relative expression levels after normalization to loading control GAPDH are presented in the lower panel. * *p* < 0.05; ** *p* < 0.01; *** *p* < 0.001.

Next, shRNA lenti viruses were transfected into PKAP+ tumor cells to silence endogenous CD34 expression, and Western blotting was used to detect the efficiency of CD34 knockdown in PKAP+ cells (Figure 4A). To examine the effects of CD34 knockdown on PDAC tumorigenesis, in vitro cell proliferation, colony forming, and wound healing assays were used to investigate and compare the differences between PKAP+ shCD34 and control PKAP+ cells. Consistently, as shown in Figure 4B, we did not observe that CD34 knockdown significantly affected PKAP+ cell proliferation. However, sphere formation assays revealed that knockdown of CD34 significantly reduced sphere formation in PKAP+ tumor cells (Figure 4C). For experimental approaches to determine the function of CD34 on PDAC cell migration, we found that knockdown of CD34 significantly reduced PKAP+ cell migration and invasion in vitro (Figure 4D,E). Furthermore, Western blotting was used to detect changes in EMT markers, kinase pathway proteins, and the CD44 cancer stemness marker in PKAP+ shCD34 cells compared to the shcontrol group. Our data revealed that shCD34 PKAP+ cells exhibited increases in p-AMPKα expression, but reduced vimentin and SMA, mesenchymal markers of EMT, and CD44 protein expression compared to sheGFP control cells (Figure 4E). Taken together, our results indicated that knockdown of CD34 inhibits cell migration and invasive ability in PDAC.

### 2.5. Overexpression CD34 Increases Tumorigenic and Cell Migration Abilities in PDAC

**Figure 5:**
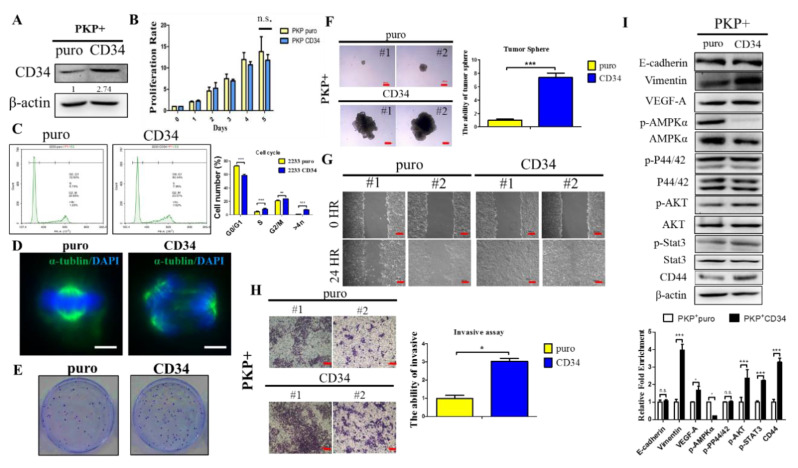
Overexpression of CD34 increases PDAC tumorigenic and migratory abilities. (**A**) Expression levels of CD34 in PKP+ pbabe-puro-CD34 and pbabe-puro control cells were determined by Western blotting analysis. (**B**) MTT cell proliferation assays showed that overexpression of CD34 does not affect tumor cell proliferation in vitro. (**C**) Analysis of cell cycle profiles by flow cytometry determined multinuclearity in CD34-overexpressed PKP+ tumor cells. (**D**) CD34-overexpressing PDAC cells exhibited greater than two centrosomes (multinuclearity) as demonstrated by costaining with anti-α-tubulin (red) antibodies and nuclear DAPI (blue). Scale bar is 50 μm. (**E**) Overexpression of CD34 in PKP+ PDAC cells enhanced colony formation according to the colony assay. (**F**) Sphere formation assays determined that overexpression of CD34 significantly increases sphere formation in PKP+ tumor cells; *p* < 0.001 compared to CD34 with pBabe puro control. (**G**) In vitro cell scratch assays revealed that overexpression of CD34 enhances cell motility in PKP+ PDAC cells. (**H**) Transwell invasion assays showed that CD34 gene overexpression promotes the invasive ability of murine PDAC cells; *p* < 0.01 compared to CD34 with pBabe puro control. (**I**) Overexpression of CD34 in PKP+ PDAC cells increased expression of p-AKT, p-STAT3, EMT markers, and CD44 protein, but downregulated the protein level of p-AMPKα according to Western blot analysis. Densitometric analysis of relative expression levels after normalization to loading control β-actin are presented in the lower panel. * *p* < 0.05; *** *p* < 0.001.

We applied the pBabe retroviral vector transfection approach to overexpress CD34 in PKP+ PDAC cells and confirmed CD34 expression by Western blotting, as shown in Figure 5A. Subsequently, we compared PKP CD34 cells and puro control PDAC cells regarding their cell proliferation, migration, and invasion abilities in vitro. Our results revealed that overexpression of CD34 in PKP cells did not affect cell proliferation significantly (Figure 5B). Fluorescence-activated cell sorting (FACS) analysis demonstrated that overexpression of CD34 in PKP+ cells resulted in increased aneuploidy cells with multinuclearity, which often results in chromosome stability and malignant development during tumorigenesis. (Figure 5C,D). Next, a tumor spheroid formation test was performed to investigate the tumorigenic ability between CD34-overexpressing PKP+ tumor cells and control cells. our results showed that overexpression of CD34 in PKP+ cells significantly increased colony formation compared to the control, as shown in Figure 5E. Meanwhile, overexpression of CD34 also promoted tumor spheroid growth in hanging drops (Figure 5F). Thus, our results revealed that overexpression of CD34 in PKP+ cells significantly increases in vitro tumorigenicity in PDAC. Further investigations regarding the effects of CD34-overexpressing PKP+ tumor cells on cell migration included the in vitro cell scratch assays, which were employed to detect their migratory abilities in vitro. We observed that PDAC cells overexpressing CD34 closed the wound within 24 h of scratching, and the wound closure rate was significantly higher than control cells (Figure 5G). Meanwhile, we also found that overexpression of CD34 in PKP cells increased cell invasion through in vitro transwell invasion analysis (Figure 5H). Furthermore, immunoblot analysis revealed that overexpression of CD34 in PDAC cells enhanced the EMT program by modulating the expression of E-cadherin and increasing expression of the mesenchymal marker vimentin in PKP+ cells (Figure 5I). Subsequently, we also observed that CD34-overexpressing PKP+ cancer cells increased p-AKT, p-STAT3, and CD44 cancer stemness protein expression, but reduced p-AMPK protein levels according to Western blot analysis (Figure 5I).

**Figure 6:**
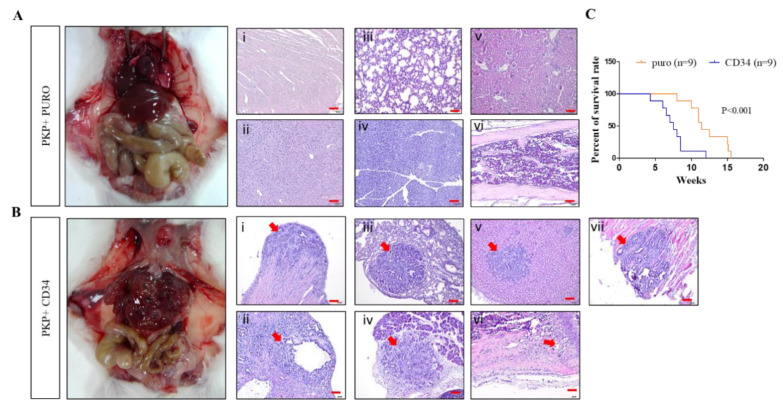
Overexpression of CD34 promotes tumor metastasis in vivo. Using the intracardiac injection approach to analyze the effect of CD34 on tumor metastasis in vivo, 2 × 10^4^ of CD34 PKP+ PDAC cells (**A**) and PKP+ control cells (**B**) were injected into the left ventricle of mouse hearts; the injected SCID mice (*n* > 3 each group) were sacrificed after three weeks. H&E-stained histological sections confirmed intensive metastasis in CD34-overexpressing PKP+-injected mice. In contrast, there were no visible metastases in the PKP+ control group. i, heart; ii, liver; iii lung; iv, pancreas; v, kidney; vi, femur bone; vii, diaphragm. Red arrows indicate metastatic tumor areas. (**C**) Kaplan–Meier curves showing the percentage of survival rates for overexpression of CD34 and puro groups. *p* < 0.001.

Given these in vitro findings, we next evaluated the prometastatic effects of CD34 on PDAC in vivo. Toward this goal, we employed intracardiac injections of CD34-overexpression PKP+ cells and control cells into severe combined immunodeficient (SCID) mice in order to evaluate their in vivo metastatic behaviors. At the end of observation (after six weeks), mice from each experimental group were sacrificed and the tumors were harvested for macro- and pathohistological examination. Our results demonstrated that the CD34-overexpressing PKP+ cell-injected mice group displayed multiple metastatic sites, with significantly shorter survival (Figure 6A,C). In contrast, we observed a significant extension of tumor-free survival in PKP+ (control)-injected mice, with no sign of any metastatic lesions in PKP+ control cell-injected animals (Figure 6B,C).

## 3. Discussion

PC has the highest mortality rate of all major cancers across the world. The five-year survival rate for people with PC is below 8% [40,41]. Notably, there are no noticeable symptoms in the early stage of PC. Certainly, symptoms may vary depending on the location of the tumor and the individual. The most common symptoms for PC include unexpected weight loss, and tumors occur in the head of the pancreas may cause jaundice and other metabolic syndromes [42]. Notably, most patients with PC are often discovered late, after PC has invaded neighboring tissues or metastasized to distant organs, so the prognosis of PC remains poor [36]. The staging of PC must be judged by computerized tomography and can be divided into four stages according to TNM, where “T” is the size of the tumor, “N” is the degree of lymph node spread, and “M” is the distant metastasis, which is the key factor that defines prognosis and is a critical element in determining appropriate treatment [38]. Distal metastasis for PC usually occurs in the liver, lungs, and peritoneal lymph. The current conventional treatment for primary PDAC is surgical resection, but because of the need to consider whether the tumor location is close to or invading the peripheral blood vessels, only about 20% of patients can undergo surgical treatment. Chemotherapy and radiotherapy represent the other current common therapeutic approaches [43]. Thus, there is an urgent need to fully understand the underlying mechanisms of pancreatic cancer metastasis and to develop better treatments in order to extend survival for patients with metastatic PC.

PDAC is the most common and aggressive form of PC. Most PDACs harbor KRAS, P16/Ink Arf, and p53 mutations; in addition, APC/β-catenin mutations have a mutation rate of 15–20% in clinical PDAC samples [11]. Recently, we reported that APC inactivation leads to increased platelet-derived growth factor (PDGF) Src signaling pathway, which increases PDAC metastasis. In this paper, we reported a metastatic PDAC animal model with activated Kras^G12D^ and p53- and APC-deficient compound mice. The loss of APC was observed to accelerate the formation of pancreatic cancer, with the metastasis rate being significantly higher than PKP model [31]. Subsequently, we isolated murine primary PDAC cells derived from Kras^G12D^, APC^L/L^, and p53^L/+^ (PKAP^+^) mice and isolated their cDNA to compare their differential gene expression profiles with PKP+ cells using cDNA microarray analysis. We focused on the increased expression of CD34 in PDAC induced by APC deletion and studied the potential roles of CD34 on PDAC development.

CD34 antigen is a cell surface glycoprotein commonly recognized as a cell surface marker for hematopoietic stem cells and is generally used for the identification and isolation of hematopoietic stem cells used for bone marrow transplantation [44]. CD34 was also reported to modulate cell–cell adhesion and enhance the migration of hematopoietic cells; these results were also observed in this paper. We found reduced Integrinβ1 expression in PKAP+ cells alongside E-cadherin expression reduction, which may led to decreased cell–cell adhesion. Additionally, CD34 functions as a stemness marker for acute myelogenous leukemia, which is a hematological malignant tumor with abnormal proliferation of bone marrow hematopoietic cells and is the most common acute leukemia in adults [45,46]. CD34+ acute myeloid leukemia stem cells are defined as CD38-negative and CD34-positive; although these subpopulations are rare, they are mainly responsible for maintaining leukemia development [47,48]. In addition, CD34+ CD38– stem cells display more resistance to chemotherapy and ultimately confer to disease recurrence as well [41]. Notably, CD34+ stem cells were also reported to play an important role in mediating liver development and regeneration [49]. Liver cancer may derive from transformed CD34+ stem cells in the liver according to multiple studies, indicating that stem cells are not only responsible for organ regeneration and tissue repair, but also represent a potential target for carcinogenesis. Additionally, isolation of CD34+ cell populations from PLC/PRF/5 hepatocellular carcinoma (HCC) can form three different histological types of liver cancer in immunodeficient mice, including hepatocellular carcinoma and hepatocellular cholangiocarcinoma (CCA), implying that CD34+ HCC cells could be a potential subtype of liver cancer stem cells (CSCs) [50].

In conclusion, this paper employed GEMMs to characterize the functional roles of APC deletion in PDAC development and found that CD34 induced by APC loss stimulated a high degree of invasion and metastasis in PDAC. Notably, our findings were further confirmed by analyzing the mRNA expression microarray dataset GSE16515, which contained 52 samples including 16 paired PDAC and normal pancreas samples and 20 PDAC samples, from NCBI GEO (http://www.ncbi.nlm.nih.gov/geo/), showing that APC negatively regulates CD34 mRNA expression in PDAC (Supplementary Figure S1). Meanwhile, according to the current experimental results, we demonstrated that increased expression of CD34 induces EMT, further promoting cell migration and metastasis of PDAC. Conversely, knockdown of CD34 inhibits PDAC invasion and migration. In summary, our study provides first-line evidence for describing the association between CD34 and the APC/Wnt pathways in PDAC and reveals the functional roles of CD34 in the progression of PDAC.

## 4. Materials and Methods

### 4.1. Genetically Modified Mice and Mouse Genotyping

Pdx-1Cre, LSLKras^G12D^, p53^Loxp/Loxp^, and APC^CKO/CKO^ mice, obtained from the Mouse Models of Human Cancers Consortium (MMHCC) under material transfer agreements, were generously made available by Drs. Andrew M. Lowy, Tyler Jacks, Anton Berns, and Raju Kucherlapati, respectively. Mice were genotyped as described by the MMHCC PCR protocols for strains 01XL5, 01XJ6, 01XC2, and 01XAA. Mice were maintained on a mixed 129SV/C57BL/6 background. Animals were maintained in the animal center at the Department of Biological Science, National Sun Yat-Sen University, under specific pathogen free (SPF) conditions and maintained in strict accordance with the principles and guidelines of the Association for the Assessment and Accreditation of Laboratory Animal Care (AAALC) for the care and use of experimental animals. All surgery and killing was performed using isoflurane or avertin to ensure minimal suffering (Institutional animal care and use committee (IACUC) approval number 10439). Pancreatic tissue samples were fixed in 10% buffered formalin overnight, washed with 1× phosphate-buffered saline, and transferred to 70% ethanol before paraffin embedding, sectioning, and hematoxylin and eosin staining.

### 4.2. Immunohistochemistry (IHC) and Immunofluorescence (IF)

H&E staining followed the standard protocol. Periodic Acid-Schiff stain (PAS) and Alcian blue staining kits were purchased from Scy-Tek Laboratories (Logan, UT, USA) and performed according to the manufacturer’s protocols. For immunohistochemistry analysis, unstained paraffin slides were baked at 56 °C overnight and deparaffinized in xylene solution twice, then rehydrated sequentially in 95%, 75%, and 40% ethanol and washed in 1× PBS. Slides were cooked for 20 min in 1× antigen retrieval buffer (H3300, Vector Laboratories, Inc., Burlingame, CA, USA), followed by 3 rinses with 1× PBS. Slides were quenched with 1% hydrogen peroxide for 10 min before incubating with blocking solution (4% normal horse serum or goat serum in PBS with 0.1% Triton X). After that, sections were incubated with primary antibody diluted in blocking solution overnight at 4 °C. Primary antibodies for anti-CD34 (HPA036722), anti-Ki67 (AB9260), and anti-ABC (05-665) were purchased from Sigma Aldrich (St. Louis, MO, USA) and Merck Millipore (Bedford, MA, USA). Slides were then washed with 1× PBS 5 times and incubated with biotinylated secondary antibody (Vector Laboratories (Burlingame, CA, USA); dilution 1:150) in blocking solution for 1 h at room temperature. After washing 5 times with 1× PBS, the slides were incubated in a Vectastain Elite ABC kit (Vector Laboratories ABC kit; PK-6100) for 30 min at room temperature. After 5 washes with 1× PBS, slides were processed for color reaction with peroxidase treatment using the 3,3′-diaminobenzidine (DAB) substrate kit (Vector Laboratories; SK-4100), washed with tap water, and counterstained with hematoxylin. Stained slides were captured using a Carl Zeiss Axioskop 2 plus microscope (Carl Zeiss, Thornwood, NY, USA) 18, 24. For immunofluorescence studies, sections were rinsed in 1× PBS after deparaffinization and incubated for 1 h at room temperature in blocking solution containing 4% normal goat or horse serum in 1 × PBS with 0.1% Triton X. Primary antibodies were diluted in blocking solution and incubated overnight at 4 °C. Subsequently, sections were rinsed in 1× PBS three times and incubated with Alexafluor-488 or Alexafluor-576 conjugated secondary antibodies (Invitrogen, Carlsbad, CA, USA). Coverslips were mounted on fluorescently labeled tissue using Vectamount with DAPI (Vector Laboratories). Images were captured using a Delta Vision Personal DV Imaging System.

### 4.3. Western Blot Analysis

Cells were harvested in 1× RIPA Buffer lysis buffer and protein concentrations were determined as previously described. Approximately 50 μg of protein was loaded and separated by SDS-PAGE, transferred to a PVDF membrane (Millipore, Danvers, MA, USA), and incubated with the following primary antibodies: anti-p-AMPKα (#2535), AMPKα (#2532), p-AKT (#9271), total AKT (#9272), phos-pP44/42 (#9101), P44/42 (#9102), p-STAT3 (#9131), total STAT3 (#9132), and N-cadherin (#4061), which were all purchased from Cell Signaling Technology (CST) Danvers, MA, USA, anti-E-cadherin(sc-7870), integrin β1 (sc-9970), vimentin (sc-373717), smooth muscle actin (SMA; sc-53142), CD44 (sc-18849), CD34 (sc-18917), and GAPDH (sc-47724) antibodies, which were all purchased from Santa Cruz Biotechnology Inc. (Santa Cruz, CA, USA), and anti-β-actin (#A1978) antibody, which was purchased from Sigma-Aldrich, St. Louis, MO, USA.

### 4.4. Real-Time–Quantitative PCR Analysis (RT–qPCR)

Total RNA prepared from samples was used for cDNA synthesis. PCR amplification was done essentially as described above, and the results of the delta CT measurements were described in detail previously [51]. Primer sequences used for real-time qPCR were as follows: mouse CD34 forward, 5′-AGGACAGCAGTAAGACCACACC-3′, and reverse, 5′-GTGTGGAGTTCCAGA GCCTGAA-3′; mouse GAPDH forward 5′-CATCACTGCCACCCAGAAGACTG-3′, and reverse, 5′-ATGCCAGTGAGCTTCCCGTTCAG-3′. These experiments were independently repeated three times.

### 4.5. Cell Proliferation Assay

For cell growth assays, 2 × 10^4^ cells were seeded in 24-well plates and incubated overnight. Cells were incubated for one to five days before 5 mg/mL MTT (thiazolyl blue tetrazolium bromide) (AMRESCO LLC, Solon, OH, USA) was added to 25 μL in 500 μL RPMI medium (Invitrogen, Carlsbad, CA, USA) and incubated for another 2 h for reaction. Medium was removed and cells treated with 200 μL DMSO (Sigma, St. Louis, MO, USA) before OD570 reading with a BioTek ELISA reader (Molecular Devices LLC, Sunnyvale, CA, USA).

### 4.6. Colony Formation and Hanging Drop Assays

Fifty thousand cells were grown in 60 mm tissue culture dishes. After 2 weeks, cells were washed with phosphate-buffered saline (PBS) and fixed with methanol and 0.1% crystal violet. The colonies were manually counted and then photographed. Spheroids were created using Perfecta3D^®^ Hanging Drop Plates (Sigma Aldrich, St. Louis, MO, USA). Spheroids of cells (2 × 10^3^ cells) were prepared and performed according to previously published protocols [51].

### 4.7. Wound Healing Assay

Cells were pretreated with 0.02% (0.2 mg/mL) mitomycin C for 2 h and wounded by removing a 300–500 mm wide strip of cells across the well with a standard 200 μL yellow tip. Wounded monolayers were washed twice with phosphate-buffered saline buffer solution to remove nonadherent cells. The cells were cultured in low FBS media and incubated for predetermined times to monitor wound closing. Wound closure was recorded by phase-contrast microscopy according to previously published protocols [51].

### 4.8. Flow Cytometry Analysis

The protocol for FACS analysis was described in detail previously. All flow cytometry analyses were performed with a BD Accuri C6 Flow Cytometer (BD Biosciences, San Jose, CA, USA) according to the manufacturer’s instructions.

### 4.9. Complementary DNA Microarray Analysis

Hybridization was performed against the Affymetrix GeneChip MoGene 1.0 ST array. The arrays were hybridized for 17 h at 45 °C and 60 r.p.m. Arrays were subsequently washed (Affymetrix Fluidics Station 450, Santa Clara, CA, USA), stained with streptavidin–phycoerythrin (GeneChip Hybridization, Wash, and Stain Kit, Affymetrix, Santa Clara, CA, USA, 900720), and scanned using an Affymetrix GeneChip Scanner 3000. The resulting data were analyzed using Expression Console software (Affymetrix) and Transcriptome Analysis Console software (Affymetrix) with default RMA parameters. The complete differential expression profiling of genes from the cDNA microarray data are summarized in Supplementary Table S1. Regulated genes were determined with a 2.0-fold change; *p*-value < 0.05

### 4.10. Murine Primary PDAC Cell Culture, Cytokine and Inhibitor Treatment

The mouse primary pancreatic cancer cells were cultured in RPMI-1640 medium supplemented with 10% fetal bovine serum, nonessential amino acids, 100 units/mL penicillin, and 100 μg/mL streptomycin at 37 °C in a 5% CO_2_ incubator. Primary mouse PDAC cells were maintained for <6 passages and histopathologically characterized through SCID mice xenograft studies before performing microarray expression profile analyses. Tumor spheroids were created using Perfecta3D^®^ Hanging Drop Plates (Sigma Aldrich, St. Louis, MO, USA). Spheroids of cells (2 × 10^3^ cells) were prepared as described above. PDAC cells were treated with 20 mM lithium chloride (LiCl) (Sigma-Aldrich 203637) for 18 h before Western blot analysis.

### 4.11. Retroviral Production and Infection of Target Cells

A retrovirus was generated by cotransfection of pBabe-eGFP empty vector or pBabe-puro-CD34 along with pVSV-G (envelope) and pVSV-GP (packaging) plasmids in 293T cells. Target cells were infected overnight with 4 mL of virus-containing medium in the presence of 10 μg/mL polybrene. The next day, cells were cultured in fresh medium and allowed to grow for another 24 h. After replacement of fresh medium, cells were selected with 2 μg/mL puromycin for 14 days; positive clones were isolated and used for further assays.

### 4.12. Lentivirus Production and shRNA for Gene Knockdown

The plasmids required for shRNA lentivirus production were purchased from the National RNAi Core Facility, Academia Sinica, Taiwan. The pLKO.1-shRNA vector used for knockdown of mouse CD34 was TRCN0000068167. The pLKO.1-shEGFP control plasmid was TRCN00000-72190 (EGFP). Lentivirus production and infection were performed according to a previously described protocol [51].

### 4.13. Intracardiac Injection of Metastatic Tumor Model

Specific pathogen-free, 8-week-old female C.B17/lcr-SCID mice were purchased from BioLASCO Taiwan Co., Ltd., Taipei, Taiwan, for the in vivo metastatic study. The animals were maintained in the animal center at the Department of Biological Science, National Sun Yat-Sen University under SPF conditions and treated according to the institutional guidelines for the care and use of experimental animals (animal permit number 10639). The B6/SCID mice (*n* = 6 each group; 20–22 g) were maintained in a well-controlled, pathogen-free environment. Intracardiac (ic) injection of prostate cancer cells was performed to allow murine PKP+ cells (CD34 versus eGFP control) to disseminate into multiple organs, including bone. Briefly, mice (>6 weeks old) were anesthetized with Tribromoethanol and murine PKP+ cells (2 × 10^4^ cells per mouse) were injected into the left ventricles of the hearts of isogenic recipient male mice. The mice were sacrificed after day 30 and all metastatic organs were collected and embedded in paraffin, sectioned, and stained with H&E, as described above.

### 4.14. Statistical Analysis

All experiments were repeated at least three times. One representative experiment was shown. RT–qPCR and cell proliferation assays were displayed as one representative experiment of three independent experiments, mean ± s.e.m. Data measured on a continuous scale were analyzed using Student’s *t*-test and categorical data were subjected to ×2 test. *p*-value < 0.05 was considered significant.

## 5. Conclusions

CD34 is predominantly expressed on hematopoietic progenitor cells and endothelial progenitors, and has been widely used as a stem and progenitor cell marker. The results of the present study provided important evidences revealing the association between CD34 and the APC/Wnt pathway in PDAC, and we demonstrated the potential roles for CD34 upregulation induced by APC inactivation in promoting PDAC cell growth, migration and invasion. Moreover, because CD34 is thought to be a biomarker for stem cells to maintain self-renewal and proper stemness, CD34 could be a novel drug candidate for the treatment of PDAC harboring APC mutations.

## Supplementary Materials

The following are available online at https://www.mdpi.com/1422-0067/21/12/4473/s1: Table S1: List of complete differential expression profiling of genes from cDNA microarray analysis in 2079 and 2545 PDAC cells; Figure S1: The increased expression of CD34 in human PDAC samples (cohort of 52 PDAC patients) was significantly related to the downregulation of APC genes.

## Abbreviations

PDAC	Pancreatic ductal adenocarcinoma
EMT	Epithelial–mesenchymal transition
IF	Immunofluorescence
IHC	Immunohistochemistry
