# Supplementary material for: Inactivation of APC Induces CD34 Upregulation to Promote Epithelial-Mesenchymal Transition and Cancer Stem Cell Traits in Pancreatic Cancer
Source: Int J Mol Sci. 2020 Jun 23;21(12):4473. doi: 10.3390/ijms21124473 (PMC7352299; doi:10.3390/ijms21124473)
Supplement: Supplementary file 1 [file ijms-21-04473-s001.zip › Supplementary Figure S1.pptx]

## Slide 1
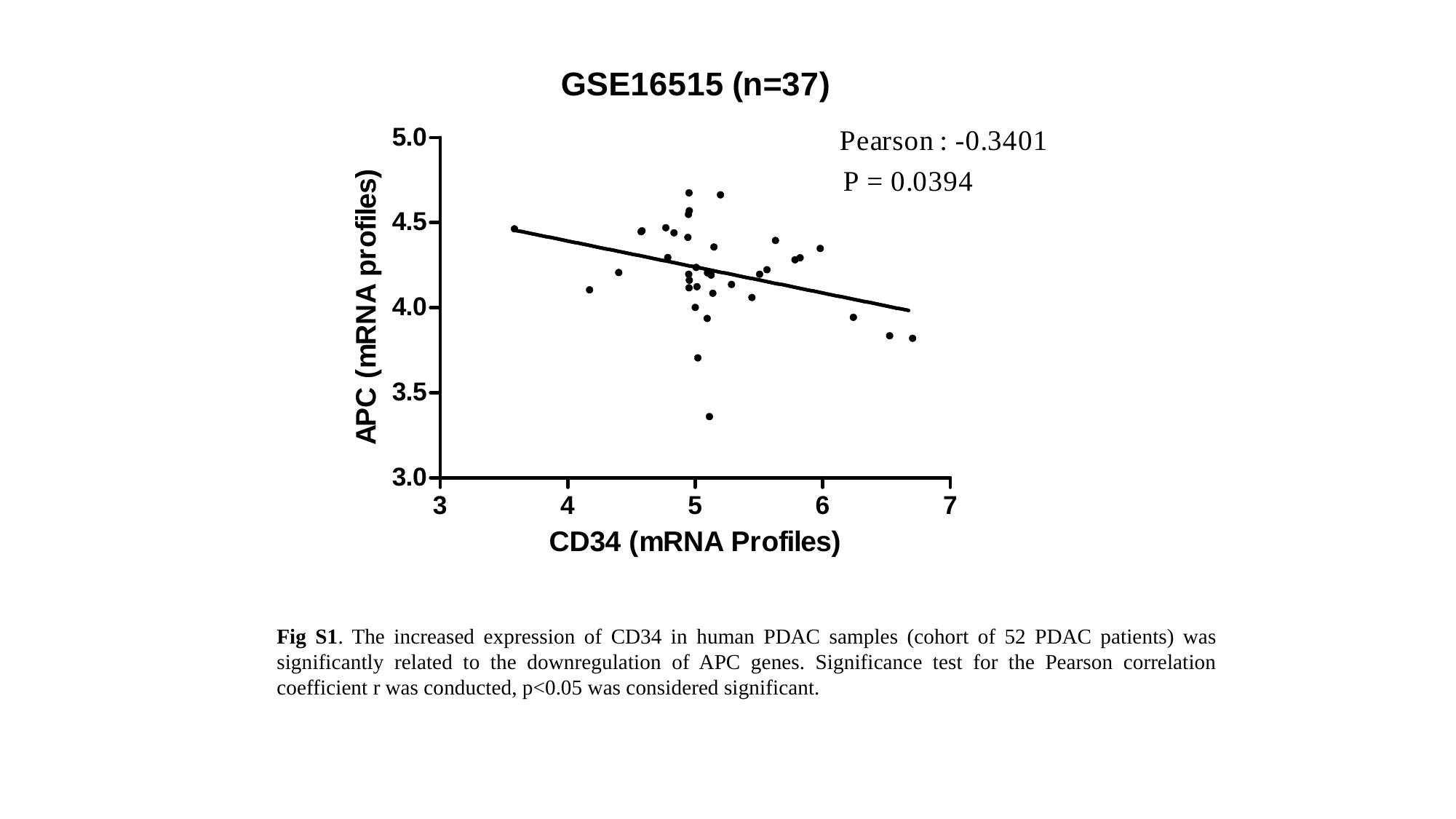

Fig S1. The increased expression of CD34 in human PDAC samples (cohort of 52 PDAC patients) was significantly related to the downregulation of APC genes. Significance test for the Pearson correlation coefficient r was conducted, p<0.05 was considered significant.
